# TDP-43/HDAC6 axis promoted tumor progression and regulated nutrient deprivation-induced autophagy in glioblastoma

**DOI:** 10.18632/oncotarget.17979

**Published:** 2017-05-18

**Authors:** Tzu-Wei Lin, Ming-Teh Chen, Liang-Ting Lin, Pin-I Huang, Wen-Liang Lo, Yi-Ping Yang, Kai-Hsi Lu, Yi-Wei Chen, Shih-Hwa Chiou, Cheng-Wen Wu

**Affiliations:** ^1^ Institute of Biochemistry and Molecular Biology, National Yang-Ming University, Taipei, Taiwan; ^2^ School of Medicine, National Yang-Ming University, Taipei, Taiwan; ^3^ Institute of Clinical Medicine, National Yang-Ming University, Taipei, Taiwan; ^4^ Department of Neurosurgery, Taipei Veterans General Hospital, Taipei, Taiwan; ^5^ Department of Medical Research, Taipei Veterans General Hospital, Taipei, Taiwan; ^6^ Cancer Center, Taipei Veterans General Hospital, Taipei, Taiwan; ^7^ Institute of Biomedical Science, Academia Sinica, Taipei, Taiwan; ^8^ Department of Health Technology and Informatics, The Hong Kong Polytechnic University, Hong Kong; ^9^ Division of Oral and Maxillofacial Surgery, Department of Stomatology, Taipei Veterans General Hospital, Taipei, Taiwan; ^10^ Institute of Pharmacology, National Yang-Ming University, Taipei, Taiwan; ^11^ Department of Neurological Surgery, Tri-Service General Hospital and National Defense Medical Center, Taipei, Taiwan; ^12^ Department of Medical Research and Education, Cheng-Hsin General Hospital, Taipei, Taiwan

**Keywords:** TDP-43, autophagy, glioblastoma, HDAC6, nutrient deprivation

## Abstract

Glioblastoma Multiforme (GBM) is a lethal primary brain tumor with poor survival lifespan and dismal outcome. Surgical resection of GBM is greatly limited due to the biological significance of brain, giving rise to tumor relapse in GBM patients. Transactive response DNA binding protein-43 (TDP-43) is a DNA/RNA-binding protein known for causing neurodegenerative diseases through post-translational modification; but little is known about its involvement in cancer development. In this study, we found that nutrient deprivation in GBM cell lines elevated TDP-43 expression by a mechanism of evasion from ubiquitin-dependent proteolytic pathway, and subsequently activated the autophagy process. Exogenous overexpression of TDP-43 consistently activated autophagy and suppressed stress-induced apoptosis. The inhibition of autophagy in TDP-43-overexpressing cells effectively increased the apoptotic population under nutrition shortage. Furthermore, we demonstrated that HDAC6 was involved in the activation of autophagy in TDP-43-overexpressing GBM cell lines. The treatment with SAHA, a universal HDAC inhibitor, significantly reduced TDP-43-mediated anti-apoptotic effect. Additionally, the results of immunohistochemistry showed that TDP-43 and HDAC6 collaborated in GBM-tumor lesions and negatively correlated with the relapse-free survival of GBM patients. Taken together, our results suggest that the TDP-43-HDAC6 signaling axis functions as a stress responsive pathway in GBM tumorigenesis and combats nutrient deprivation stress via activating autophagy, while inhibition of HDAC6 overpowers the pathway and provides a novel therapeutic strategy against GBM.

## INTRODUCTION

Glioblastoma (GBM), classified as Grade IV brain tumor by World Health Organization (WHO), is the most common and aggressive malignant primary brain tumor [1]. GBM is a highly differentiated, vascularized and diffusive tumor which is clinically difficult since surgical resection is the major strategy for the therapy [2]. However, the diffusive nature of GBM makes the boundary between normal brain tissues and GBM hard to define, resulting in clinical difficulty to remove the tumor. As a result, the remnant GBM cells would eventually contribute to GBM recurrences. Therefore, understanding the biomolecular mechanisms of GBM progression and its development of drug resistance to current therapies is critical to improve GBM treatment.

TDP-43, also known as transactive response (TAR) DNA binding protein-43 (TARDBP), belongs to heterogeneous nuclear ribonucleoprotein (hnRNP) family and is a well-characterized RNA-binding protein (RBP). TDP-43 has been closely linked to the pathogenesis of neuronal degenerative diseases, including amyotrophic lateral sclerosis (ALS) and frontotemporal lobar degeneration (FTLD) upon post-translational modification [3]. In response to various stressful conditions, TDP-43 forms and modulates stress granules via regulating G3BP and TIA-1 [4]. Despite a few reports revealing the relationship between TDP-43 and gliomas, little is known about the role of TDP-43 in tumor malignancy and progression [5, 6]. Besides, recent studies indicated that TDP-43 participates in regulation of glucose and lipid metabolism [7–9]. TDP-43 was also linked to the metabolic consumption through regulating miR-520-associated glycolysis in hepatocellular carcinoma [10].

Nutrient deprivation is often observed in solid tumors due to the rapid growth rate and ineffective blood supply in tumor tissue. Autophagy has been implicated as the key regulatory mechanism for cells to escape from cytotoxic stress. The role of autophagy in cancer therapy has been implicated to against cytotoxic, plenty of clinical trials have been launched targeting the inhibition of autophagy while the expression of autophagy-associated proteins, such as beclin-1 and LC3, are also connected with the poor prognosis of cancers [11–13]. The role of TDP-43 in autophagy mechanism was also investigated but it was limited to the cases of neurodegenerative diseases rather than malignant neoplasms [14]. Here we present the potential mechanism of GBM therapeutic resistance, by which TDP-43 directs activation of LC-3 in response to nutrient deprivation, and thus protects the cancer cells from apoptotic death. Metabolic dysregulation is one of the cancer hallmarks, and cancerous cells reorganize metabolic pathways to adapt to nutrient deprivation. The phenomenon, known as Warburg effect, allows cancer cells to predominantly generate energy through glycolysis rather than TCA cycle [15]. Increased metabolic demand for ATP starves the cells, and therefore causes metabolic stress. In meanwhile, the rapid growth of tumor causes partial hypoxia and nutrient deprivation in the core of tumor mass due to insufficient blood supply. These metabolic stresses activate self-cannibalization mechanism of autophagy in cancer cells, resulting in lysosomal turnover, the recycling of cell organelles, and eventually this allows cells to survive. Once the oncogenic process is activated, a protective mechanism activates cellular recycling and adapts cells to insufficient nutrient supplement [16, 17]. Induction of acute metabolic stress has a well-known anti-cancer effect, which allowed to develop a number of anti-cancer drugs that eliminate the ability of cancer cells to overcome metabolic stress for survival. However, the efficiency of such drugs is compromised by induction of autophagy process in cancer cells, as has been reported for Temozolomide (TMZ) [18, 19]. As a result, autophagy promotes degradation of misfolded proteins and recycling of cellular substrates, thus promoting cancer cell survival and tumor growth.

In this study we investigated the mechanism of how TDP-43 promotes survival of the cells upon nutrition deprivation and contributes to the progress of GBM. We found that elevated expression of TDP-43 activates autophagy in stressful conditions and rescues cells from apoptotic process. Immunoprecipitation of TDP-43 revealed the increase of ubiquitination indicating the shortening of protein half-life of TDP-43 during starvation. Moreover, we discovered that HDAC6 acts as a downstream effector of TDP-43 in response to nutrient deficiency. In meanwhile, the inhibition of HDAC increases the apoptotic events even when TDP-43 is overexpressed. Concomitantly, we compared our findings to the clinical data and found that TDP-43 and HDAC6 are co-expressed and their high-expression reflected the better survival than that of others.

## RESULTS

### TDP-43 expression in human glioblastoma cell lines increases their tumorigenic properties

**Figure 1:**
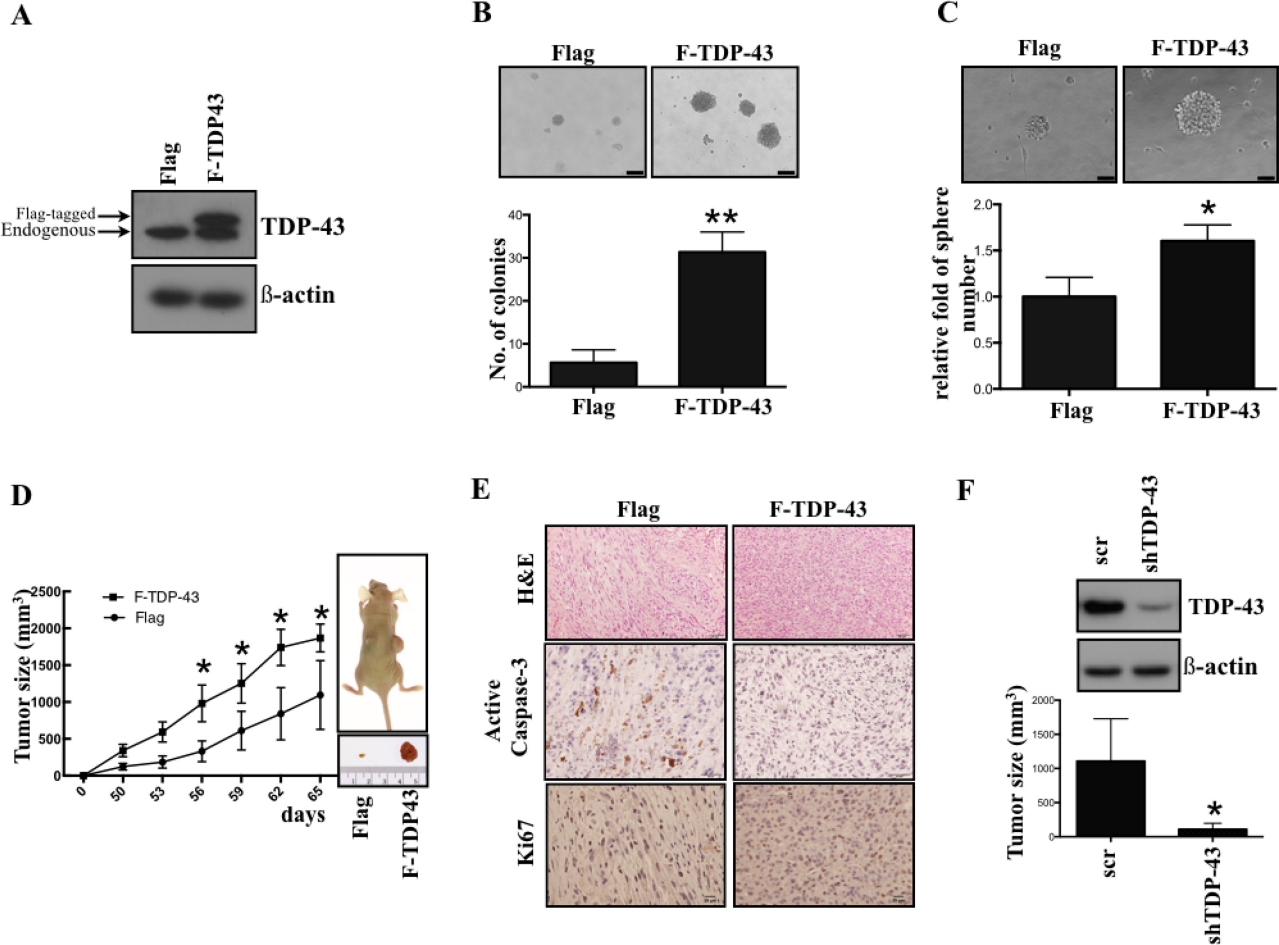
TDP-43 is essential for tumor progression *in vitro* and *in vivo* (**A**) TDP-43 protein expression in GBM cell lines. Total cell lysates of indicated cells were subjected to Western blotting, and detected by TDP-43 specific antibody. β-actin was used as a loading control. Flag, vector control; F-TDP-43, Flag-tagged TDP-43. (**B**) Colony formation of U87MG cells. U87MG cells Flag or Flag-TDP-43 stably over-expressing were transferred to agar-coated plate and incubated for 4 weeks. Colonies were stained by crystal violet. The number of colonies was counted and quantitative data are shown as mean±SD (lower). (**C**) Sphere formation of U87MG cells. U87MG cells stably over-expressing TDP-43 were incubated in neural induction medium for 4 weeks. The relative fold of sphere are shown (lower) (**D**) Growth curves of xenograft tumors in nude mice implanted with U87MG cells stably over-expressing Flag or Flag-TDP-43, respectively. Tumor growth was measured weekly. (**E**) Immunochemistry of U87MG xenograft tumor. Serial sections from xenografts derived from U87MG stably over-expressing TDP-43 or control xenograft were stained with H&E stain, active caspase-3 or Ki67 specific antibody. (**F**) Xenografts of U87 scramble and shTDP-43 cells. U87MG cells transfected with shTDP-43 were subtaneuously injected into nude mice. Tumor formation was measured in 12 weeks.

In order to elucidate the role of TDP-43 during tumorigenesis, we first introduced Flag-tagged TDP-43 into U87MG GBM cell line to generate stable TDP-43-over-expressing cell line. The expression of TDP-43 was examined by Western blotting (Figure 1A). To determine whether TDP-43 is associated with GBM stem cell (GSC) property, the soft agar assay for colony formation and suspended sphere formation assay for self-renewal state were performed to determine the anchorage independent growth ability of U87MG cells. Significant increase of the colony formation was observed in the TDP-43-overexpressing cells (Figure 1B, *p <* 0.01), while TDP-43 also enhanced the formation of neurospheres of U87MG (Figure 1C, *p <* 0.05). To further assess the role of TDP-43 in tumorigenicity, we subcutaneously implanted the TDP-43-overexpressing cells into nude mice. In comparison to the empty vector control (Flag-control), the size of tumors was greatly increased in Flag-TDP-43 group (Figure 1D). In meanwhile, the immunohistochemistry (IHC) staining demonstrated that overexpression of TDP-43 repressed expression of pro-apoptotic caspase 3 and also increased the pro-proliferative Ki-67 expression (Figure 1E). Using short hairpin RNA (shRNA), we repressed expression of endogenous TDP-43 in U87MG cell line, which resulted in tumor size reduction in xenotransplanted mice (Figure 1F). Taken together, these data support the oncogenic role of TDP-43 in GBM tumorigenesis and growth induction.

### TDP-43 protects glioblastoma cells from nutrient deprivation-induced cell death

**Figure 2:**
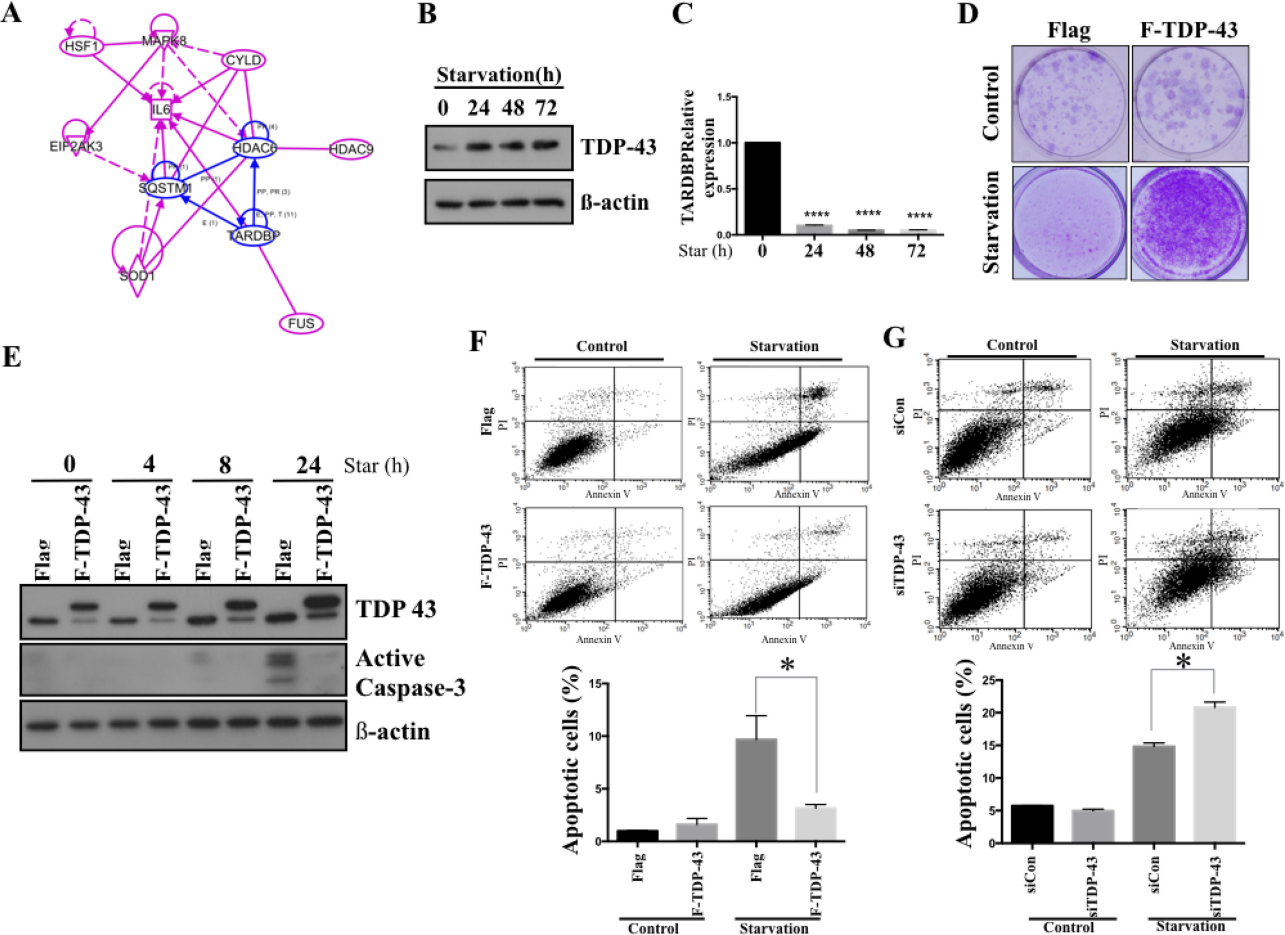
TDP-43 protects glioblastoma cell from nutrition deprivation-induced cell death (**A**) Ingenuity Pathway Analysis (IPA) of TDP-43 and Warburg effect-associated genes. (**B**) Protein expression of TDP-43 in starved cells. U87MG cells were treated with HBSS for indicated times. After starvation, total cell lysates were subjected to Western blotting analysis using TDP-43 specific antibody. β-actin was used as a loading control. (**C**) RNA expression of TDP-43 in U87MG cells. Total RNA from starved cells was collected and TDP-43 expression was quantified by quantitative PCR. 18S was used to nornalize RNA concentration. (**D**) Clongenic assay of U87MG cells. Flag, vector control; F-TDP-43, Flag-tagged TDP-43 stably over-expressing U87MG cells. In starvation group, After 6days starvtion, we refresh U87MG cells with fresh medium and culture for 14 days. (**E**) Western blotting of active Caspase-3 under starvation. U87MG cells stably expressing TDP-43 or Flag control were nutrient-deprived for indicated time and total cell lysates were subjected to Western blotting analysis using TDP-43 or Caspase-3 specific antibody. β-actin was used as a loading control. (**F** and **G**) Apoptotic fraction of cells was detected by annexin V/PI staining. Cells with (F) TDP-43 overexpression or (G) silencing of TDP-43 were starved for 24 hours and stained by annexin V/PI. Apoptotic cells were measured by flow cytometry.

Limited vascularisation caused by rapid tumor growth restricts local oxygen and nutrient supply and results in a stressful condition. However, the malignant neoplasms, including GBM, are able to survive this stress, although little is known about the detailed molecular mechanisms supporting such survival. Warburg effect is one of the most well-established mechanisms of tumor survival under nutrient shortage. In an *in silico* analysis using knowledge-based Igenuity Pathway Analysis (IPA, Qiagen), the TDP-43 was found to be involved in Warburg effect-associated pathways (Figure 2A). Nutrient deprivation causes increase of TDP-43 expression in a time-dependent manner, which suggests that the expression of TDP-43 is possibly induced by metabolic stress (Figure 2B, 2C). In addition, the colony-forming ability of parental cells was limited upon nutrient deprivation, which could be eventually restored by exogenous over-expression of TDP-43 (Figure 2D). We also found that TDP-43 expression in U87 GBM cells correlated with nutrient deprivation-induced apoptosis. Under the nutrient deprivation condtions, ectopic expression of TDP-43 down-regulated caspase 3 activation (Figure 2E). In meanwhile, ectopic TDP-43 expression decreased the nutrient deprivation-induced apoptosis in GBM cells, as compared with the control. (Figure 2F, *p <* 0.05). In contrast, siTDP-43 enhanced the starvation-induced proportion of apoptotic cells in U87MG as compared to scrambled siRNA control (Figure 2G, *p <* 0.05). These data indicate that TDP-43 protects GBM cells from nutrient deprivation-induced cell death.

### TDP-43 protects GBM cells from nutrient deprivation by promoting autophagy

**Figure 3:**
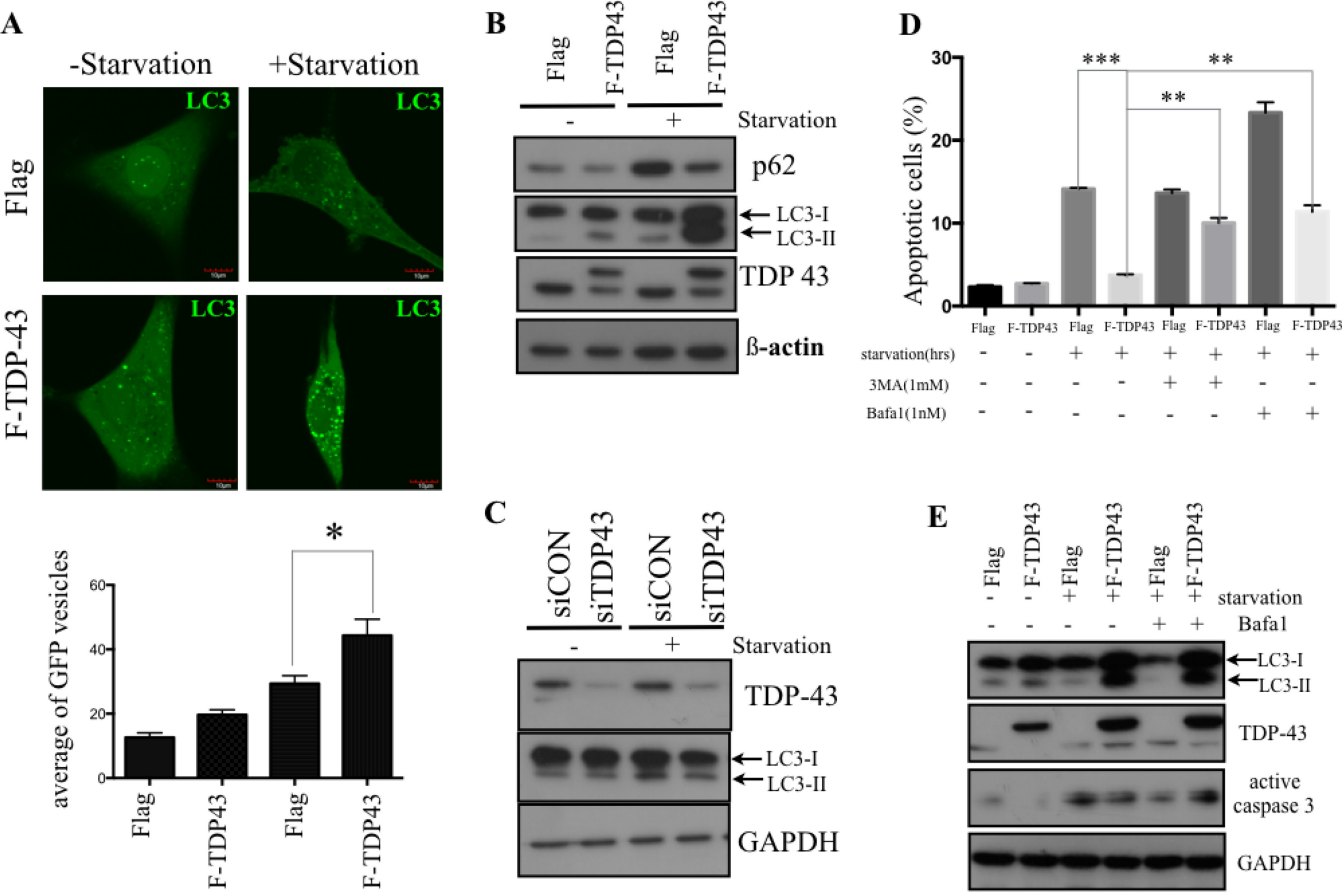
TDP-43 promotes autophagy formation under nutrient deprivation (**A**) Autophagosome formation in starved cells. U87MG cells stably over-expressing TDP-43 or Flag control were transfected with GFP-LC3 and were grown in either complete medium or nutrient-deprived medium. Autophagosome formation was analyzed by flourescent microscopy. Quantitative data are expressed as the numbers of GFP punctates in a cell (mean±SD, lower). (**B** and **C**) Western blotting of LC3 expression. Cells starved for indicated times were subjected to Western blotting using P62, LC3 and TDP-43 specific antibodies. β-actin was used as a loading control. (**D**) Apoptotic cells under nutrient deprivation. U87MG cells stably expressing TDP-43 or Flag control were treated with BafA1 (0.5 nM) or 3-MA (1 mM), respectively, and were cultured with or without starvation for 24 hours. After starvation, cells were stained with annexin V/PI and measured by flow cytometry. (**E**) Western blotting of active caspase 3 in U87MG. Cells treated with DMSO, Bafa1 (0.5 nM) were starved for the 24 hours, and were collected and subjected to Western blotting using LC3, TPD-43, Caspase-3 specific actibodies. GAPDH was used as internal control.

Autophagy has been reported to contribute to cancer chemoresistance and metabolic regulation [20]. To futher evaluate the effect of TDP-43 on autophagy in GBM, we examined the correlation between TDP-43 expression and autophagosome formation in GBM cells under nutrient deprivation by transfecting GFP-tagged autophagosome protein LC3 to U87MG cells. Stable over-expression of TDP-43 significantly increased formation of autophagosomes under nutrient depriviation (Figure 3A, *p <* 0.05). Western blotting further indicated that the endogenous autophagy marker LC3-II accumulated in nutrient-deprived U87MG cells with TDP-43 over-expression. (Figure 3B). On the other hand, knockdown of TDP-43 cells decreased LC3 II protein level in nutrient-deprived U87MG cells comparing to scrambled control cells (siCON) (Figure 3C).

Next, in order to assess the crosstalk between autophagy and starvation-induced cell death, we treated U87 cells with inhibitors of autophagy BafA1 or 3-MA under nutrient deprivation conditions. The proportion of apoptotic cells was measured by flow cytometry using annexin V/PI staining. In accordance with Figure 2, TDP-43 over-expression decreased the proportion of apoptotic cells under nutrient deprivation. However, this anti-apoptotic effect was significantly reduced in TDP-43-overexpressing treated with Bafa1 or 3-MA (Figure 3D) The anti-apoptotic effects were also examined by Western blotting through detecting activated caspase-3 (Figure 3E). Treatment of U87MG cells with autophagy inhibitor BafA1 resulted in increase of active Caspase-3, thus indicating to role of autophagy in TDP-43-mediated anti-apoptotic effect. To summarize, these data indicate that TDP-43 parcitipates in autophagosome formation under nutrient deprivation, promoting the escape from programmed cell death.

### Nutrient deprivation enhances TDP-43 protein stability by inhibiting its degradation by proteasome

**Figure 4:**
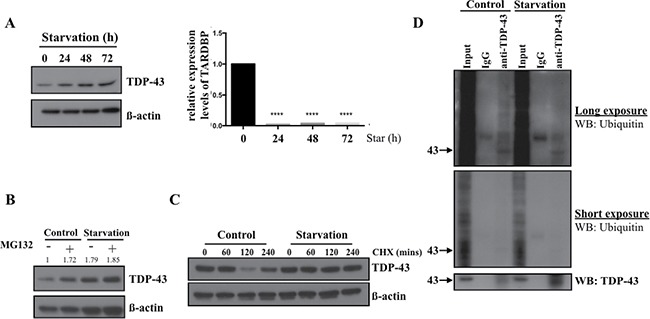
TDP-43 degradation is inhibited in nutrient deprivation condition (**A**) TDP-43 expression under starvation. 05MG cells were starved for indicated time and total cell lysates were subjected to Western blotting using TDP-43 specific antibody. β-actin was used as a loading control. The mRNA level of TDP-43 under starvation. U87MG cells starved for indicated time were collected and total RNA was analyzed by quantitative PCR. 18S was used as internal control. (**B**) Protein expression of TDP-43 under starvation condition. 05MG cells were nutrient-deprived for 8 hours with or without 25uM MG132. Expression of TDP-43 in total cell lysates was analyzed by Western blotting using an anti-TDP-43 antibody. β-actin was used as a loading control. (**C**) U87MG cells were treated with DMSO or CHX (50 um) for indicated time in starvation (HBSS) or complete medium. TDP-43 expression was analyzed by immunoblotting using TDP-43 specific antibody. β-actin was used as a loading control. (**D**) Ubiquitination of TDP-43 in starved cell. Total cell lysates from nutrient-deprived cells or control cells were immunoprecipitaed with TDP-43 antibody and subjected to Western blotting. The signals were detected by ubiqunitin or TDP-43 specific antibody.

To explore the mechanism of increased TDP-43 expression in starved GBM we used 05MG cells, which, similarly to U87MG cells, demonstrated increased TDP-43 levels in response to starvation, (Figure 4A). Next we tested whether enhanced TDP-43 expression is caused by increase of mRNA transcription or increased protein stability. Quantitative real-time PCR was performed to measure the mRNA level of TDP-43 following nutrient deprivation. The level *TDP-43* mRNA in nutrient-deprived 05MG cells was of significantly decreased from 24 to 72 hours of starvation (Figure 4A), which implied that increase of TDP-43 protein level was not due to induction of transcription in starvation conditions. Considering that TDP-43 was previously reported to be degraded via the proteasome-related pathway, we tested the hypothesis that TDP-43 stability is enhanced during starvation by evading proteasome-dependent degradation by treating cells with the proteasome inhibitor MG132. As a result, MG132 significantly increased TDP-43 accumulation in complete medium, but not in nutrient-deprived medium (Figure 4B). This data indicated that increase of TDP-43 in nutrient-deprived cells was possibly due to inhibition of proteasome degradation. On the other hand, cycloheximide (CHX), a chemical compound that blocks protein synthesis, was used for analyzing protein stability of TDP-43. CHX treatment significantly reduced TDP-43 protein levels in 05MG cells in a time-dependent manner, however, no significant inhibitory effect was observed in the nutrient-deprived condition, suggesting that nutrient deprivation sustains TDP-43 protein stability and prevents proteasome-dependent degradation of TDP-43 (Figure 4C). Given the fact that proteasome degradation is tightly regulated by ubiquitination proteasome system (UPS), we performed ubiquitin immunoprecipitation assay to determine any change of ubiquitination status of TDP-43 upon starvation. For this purpose, we immunoprecipitated the whole cell lysates with anti-TDP-43 antibody and analyzed immunoprecipitates by Western blotting using anti-ubiquitin antibody. As shown in Figure 4D, the signal of ubiquitination was reduced upon nutrient deprivation. Collectively, these data imply that TDP-43 protein stability is enhanced in the condition of nutrient deprivation as a consequence of evasion from the ubiquitination/proteasome-dependent proteolysis.

### Expression of TDP-43 in starved U87MG cells is associated with enhanced HDAC6 expression that is required for TDP-43-mediated anti-apoptosis

**Figure 5:**
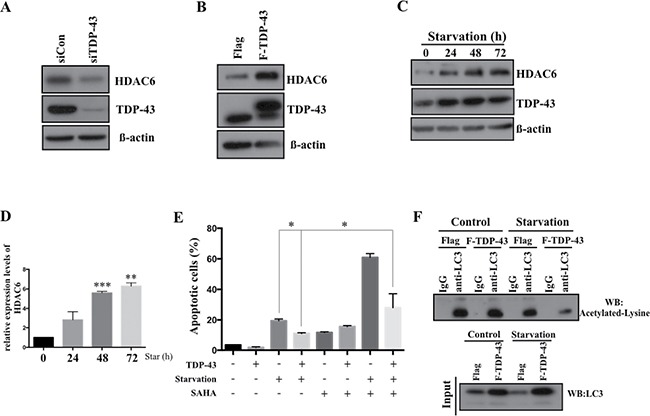
TDP-43-HDAC6 axis mediates autophagy-dependent anti-apoptosis effect HDAC6 expression in U87MG cells. TDP-43 was down-regulated by (**A**) siTDP-43 or (**B**) overexpressed in U87MG cells. Total cell lysates were subjected to Western blotting using HDAC6 or TDP-43 specific antibody. β-actin was used as a loading control. (**C**) TDP-43 and HDAC6 expression in starved U87MG. U87MG cells were nutrient-deprived for indicated times. After deprivation, total cell lysates were subjected to Western blotting using HDAC6 and TDP-43 specific antibodies. β-actin was used as a loading control. (**D**) Quantitative real time PCR analysis of HDAC6 expression. Data are represented as mean ± S.D. (**E**) Apoptotic cells under nutrient deprivation. U87MG cells stably over-expressing TDP-43 or control cells were treated with DMSO or SAHA (50 uM) and cultured with or without nutrient deprivation. Apoptotic cells were detected by annexin V/PI staining using flow cytometry. (**F**) Acetylated LC3 in nutrient-deprived cells. FLAG- or FLAG-TDP-43-expressing U87MG cells were cultured with or without nutritent deprivation. LC3 was immunoprecipated and subjected to Western blotting using acetylated lysine specific antibody.

Given the fact that TDP-43 is an RNA/DNA binding protein involved in transcriptional repression and mRNA processing, and autophagy has previously been found to be regulated by histone deacetylase 6 (HDAC6) as a downstream effector of TDP-43 [21], we expected a critical role of HDAC6 expression in U87MG cell line during nutrient deprivation. Consistently with the previous findings, loss of TDP-43 down-regulated HDAC6 expression (Figure 5A) and ectopic induction of TDP-43 enhanced the expression of HDAC-6 (Figure 5B). Furthermore, we found that expression of HDAC6 was significantly increased in nutrient-deprived U87MG cells. Moreover, nutrient deprivation caused a concurrent increase of TDP-43 and HDAC6 expression (Figure 5C, 5D), of which the expression of HDAC-6 was up-regulated in a time-dependent manner. Next, we aimed to elucidate whether TDP-43/HDAC6 axis contributes to anti-apoptotic effect in nutrient-deprived cells. We treated U87MG cells with SAHA, a universal HDACs inhibitor, under nutrient deprivation. Strikingly, TDP-43-mediated anti-apoptotic effect was reduced upon SAHA treatment (Figure 5E). Deacetylation of LC3-II in nutrient deprivation which involves in autophagy formation is regulated by HDAC6. LC3 was immunoprecipitated by LC3 specific antibody and subjected to Western blotting for detecting acetylation of LC3. In stably TDP-43 expressed U87MG cells. Acetylated LC3-II was significantly reduced under nutrient deprivation (Figure 5F). These data further strengthen that TDP-43/HDAC6 axis participated in autophagy mediated anti-apoptosis effects. In conclusion, expression of TDP-43 in GBM causes HDAC6 upregulation and might thereby contribute to anti-apoptosis.

### TDP-43 and HDAC6 correlate with poor survival outcome in brain tumor patients

**Figure 6:**
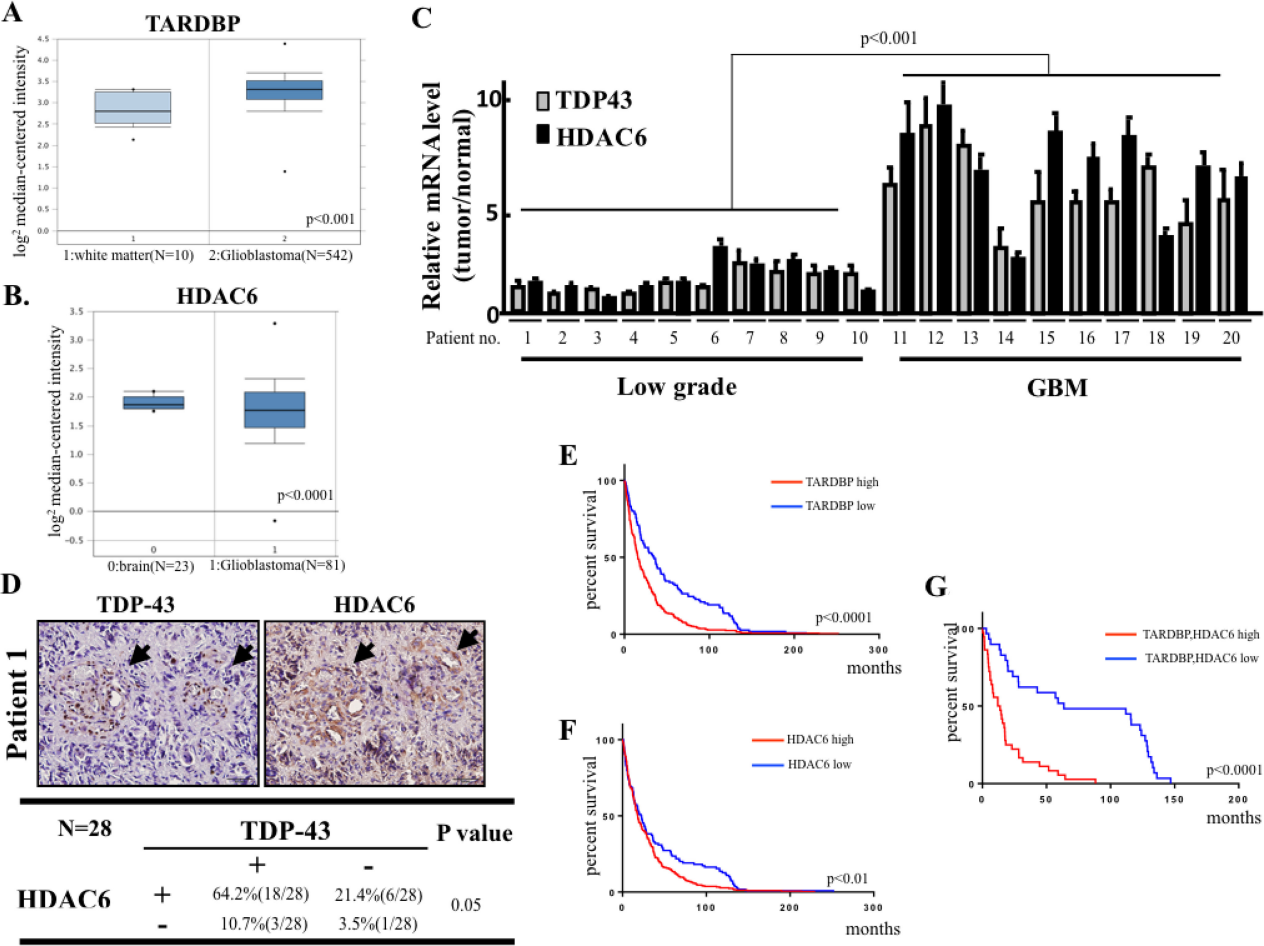
Clinical significance of TDP-43/HDAC6 protein expression and survival of human brain tumors patients (**A**) Box plot showing expression of TDP-43 (*TARDBP*) mRNA in TCGA microarray dataset consisting of white matter (normal, *N* = 10) and GBM (*N* = 542) samples. (**B**) Microarray data for HDAC gene expression from Sun Brian dataset consisting of normal, *N* = 23) and GBM (*N* = 81) samples. (**C**) The total RNA from 10 low-grade glioma and 10 GBM high-grade tumors was analyzed for TDP-43 and HDAC6 mRNA expression by quantitative real-time PCR. (**D**) The protein expression of TDP-43 and HDAC6 was analyzed by immunohistochemical (IHC) staining analysis in GBM tumor samples. Chi-square test was used for statistical analysis. (**E**) The Kaplan-Meier survival curve for brain tumor patients with differential TDP-43 (*TARDBP*) expression according to Rembrandt database (TARDBP high, *N* = 327, TARDBP low, *N* = 110). (**F**) The Kaplan-Meier survival curve for brain tumor patients with differential HDAC6 expression according to Rembrandt database (HDAC6 high, *N* = 327, HDAC6 low, *N* = 110). (**G**) Kaplan-Meier analysis of the survival in TARDBP and HDAC6 co-expression in brain tumor patients (TAARDBP/HDAC6 high, *N* = 29, TARDBP/HDAC6 low, *N* = 36).

Since our data suggested that TDP-43 and TDP-43/HDAC6 axis promotes GBM-related tumorigenesis, we therefore examined the expression patterns of TDP-43 and HDAC6 in GBM patient samples. First, we analyzed microarray dataset from TCGA Brain (Oncomine database) and found that expression levels of TDP-43 were significantly elevated in GBM specimens in comparison with the clinical white matter (normal brain) (Figure 6A). Secondly, the comparative data from Sun Brain dataset (Oncomine database) indicated that HDAC6 was expressed at higher levels in clinical GBM specimens than in clinical normal brain tissue (Figure 6B). Furthermore, we investigated whether TDP-43 and HDAC6 were dysregulated in a correlated manner in malignant glioma. The brain tumor samples from ten low-grade glioma patients and ten GBM patients were analyzed by quantitative real-time PCR to measure the expression levels of TDP-43 and HDAC6 mRNAs. The analysis revealed that mRNA levels of both TDP-43 and HDAC6 were significantly higher in GBM samples than in low-grade glioma samples (*p <* 0.001; Figure 6C). Notably, our results indicated TDP-43 and HDAC6 mRNAs were co-expressed in the GBM samples. To further validate the protein-interacted relationship between TDP-43 and HDAC6 in GBM-patient samples, a correlational study carried out in 28 sample showed significant correlations between TDP-43 and HDAC6 expression. The positive signal of TDP-43 and HDAC6 were scored into four group according to the intensity of staining signal (0, without signal; 1, weakly; 2, moderately signal; 3, strong signal). The 0 and 1 were classified into negative group, 2 and 3 were classified into positive. (Figure 6D). Collectively, these clinical results further strengthened the correlation between TDP-43-HDAC6 pathway and GBM progression. To evaluate the correlation of TDP-43 or HDAC6 expression with patient prognosis, Kaplan-Meier survival curves have been generated from The Repository of Molecular Brain Neoplasia (REMBRANDT) database (Figure 6E–6G). We found that the patients with high expression of TDP-43 and HDAC6 had worse survival rate than the group with low expression of TDP-43 or HDAC6. Notably, the survival of GBM patients was explicitly correlated with the protein signature of TDP-43^hi^/HDAC6^hi^. Collectively, we demonstrated that the co-elevated expression of TDP-43 and HDAC6 positively correlated with the high-grade glioma and GBM, suggesting the TDP-43/HDAC6 could be a potential marker of prognosis in GBM.

## DISCUSSION

Understanding of the molecular mechanisms of tumorigenesis in glioblastoma multiforme (GBM) is crucial for developing the effective therapeutic approaches and improving patient survival. In this study, we focused on expression of the nutrient deprivation-induced TAR DNA-binding protein (TDP-43) and its downstream effector histone deacetylase-6 (HDAC6) to dissect their involvements in tumorigenesis of GBM. Our results indicate that TDP-43 expression is essential for tumor progression (Figure 1) by promoting autophagy that protects cells from stress-induced cell death in the conditions of nutrient deprivation (Figures 2 and 3). The up-regulation of TDP-43 in starved cells was shown to be due to inhibition of its degradation by proteasome-dependent mechanism (Figure 4). Overexpression of TDP-43 and HDAC6 in the late stage GBM patients raised the possibility that expression levels of TDP-43 and HDAC6 could be induced by cellular stress triggered by therapeutic treatment or nutrient deprivation that is common to highly malignant and advanced cancer cells (Figures 5 and 6). Moreover, autophagy is a self-degenerative mechanism that is crucial to maintain cellular homeostasis [22]. In tumor, autophagy is regarded as a survival-promoting pathway that is required for tumors to overcome the critical microenvironment [20, 23]. Previous study reported that TDP-43 binds to 3′UTR of *ATG7* mRNA that encodes an autophagy regulating factor, which is required to maintain autophagy system [24]. Notably, TDP-43-mediated autophagy was found in this study to mitigate nutrient deprivation-induced apoptosis and lead to tumor progression, which is highly consistent with our data that TDP-43 is important for tumor survival under metabolic stress.

TDP-43 has been reported to be involved in repression of HIV transcription and regulation of alternative splicing of CFTR gene [25–27]. Because of the correlation between TDP-43 and neurodegenerative diseases, most studies were focused on its role in neuronal diseases. Abnormal aggregation of TDP-43 and its fragments is known as a toxic factor in neurons subjected to neurodegeneration [28, 29]. Abnormalities of TDP-43 are highly correlated with neurodegenerative diseases, including amyotrophic lateral sclerosis (ALS) and frontotemporal lobar degeneration (FTLD) [3]. Translocation of TDP-43 from the nucleus to mitochondria results in mitochondrial dysfunction, which is a key pathological feature of neurodegenerative diseases, including Alzheimer’s disease, Parkinson’s disease and Huntington’s disease [30–34]. Although TDP-43 is ubiquitously expressed and participates in many cellular functions, such as apoptosis and glucose homeostasis [35, 36], the physiological functions of TDP-43 are still unclear. Nutrient deprivation has been widely applied for anti-cancer treatment in a variety of approaches, such as cutting off blood supply by surgical ligation, embolization, and anti-angiogenic agent administration. However, some studies reported that the shortage of nutrition uptake may increase the drug insensitivity rather than tumor mass shrinkage, probably as a result of activation of autophagy [37–39]. In this study, we have shown that stability of TDP-43 is elevated under nutrient deprivation, suggesting a potential role of TDP-43 as a metabolic stress responsive factor. Nutrient deprivation exists in solid tumors due to poor blood supply, therefore TDP-43 is possibly responsible for tumor survival in the center of GBM tumor mass, which is poorly supplied with oxygen and nutrients.

Histone deacetylases (HDACs) are involved in epigenetic modification through histone deacetylation. Eighteen HDACs were identified in mammals, among them class I HDAC family members that include HDAC1 and HDAC2, are localized in the nucleus and regulate some biological events in cancer cells, including apoptosis and proliferation. Unlike type I HDACs, HDAC6 is categorized as type IIB HDAC and is mostly located in the cytoplasm, where it is predominantly engaged in cytoskeletal regulation [40, 41]. HDAC6 targets tubulin, HSP90, and cortactin, to remodel the microtubule formation, as well as cytoskeleton dynamics, and subsequently affects cell mobility [42–44]. In addition to acceleration of cell migration, HDAC6 was reported to promote tumorigenesis and metastasis through regulating ubiquitin and tubulin [45, 46]. Since then, HDAC6 has become a target for cancer therapy due to its pivotal role in oncogenic cellular transformation [47, 48]. Most recent study has revealed that HDAC6 can promote proliferation and drug resistance in GBM treated with Temozolomide [49]. Currently, several clinical trials have been launched using analogues of Trichostatin A (TSA), the universal HDAC inhibitors, such as Vorinostat (SAHA), Belinostat (Beleodaq) and Abexinostat (PCI-24781). [50]. However, the non-selective inhibition of HDACs may result in severe side effects, so the development of specific inhibitor of HDAC6 is of utmost importance [51, 52]. To the best of our knowledge, this is the first report demonstrating that TDP-43/HDAC6 regulation plays critical role in the progression of glioblastoma. We propose that specific HDAC6 inhibitor could be a potent therapeutic target for treating GBM in the future.

**Figure 7:**
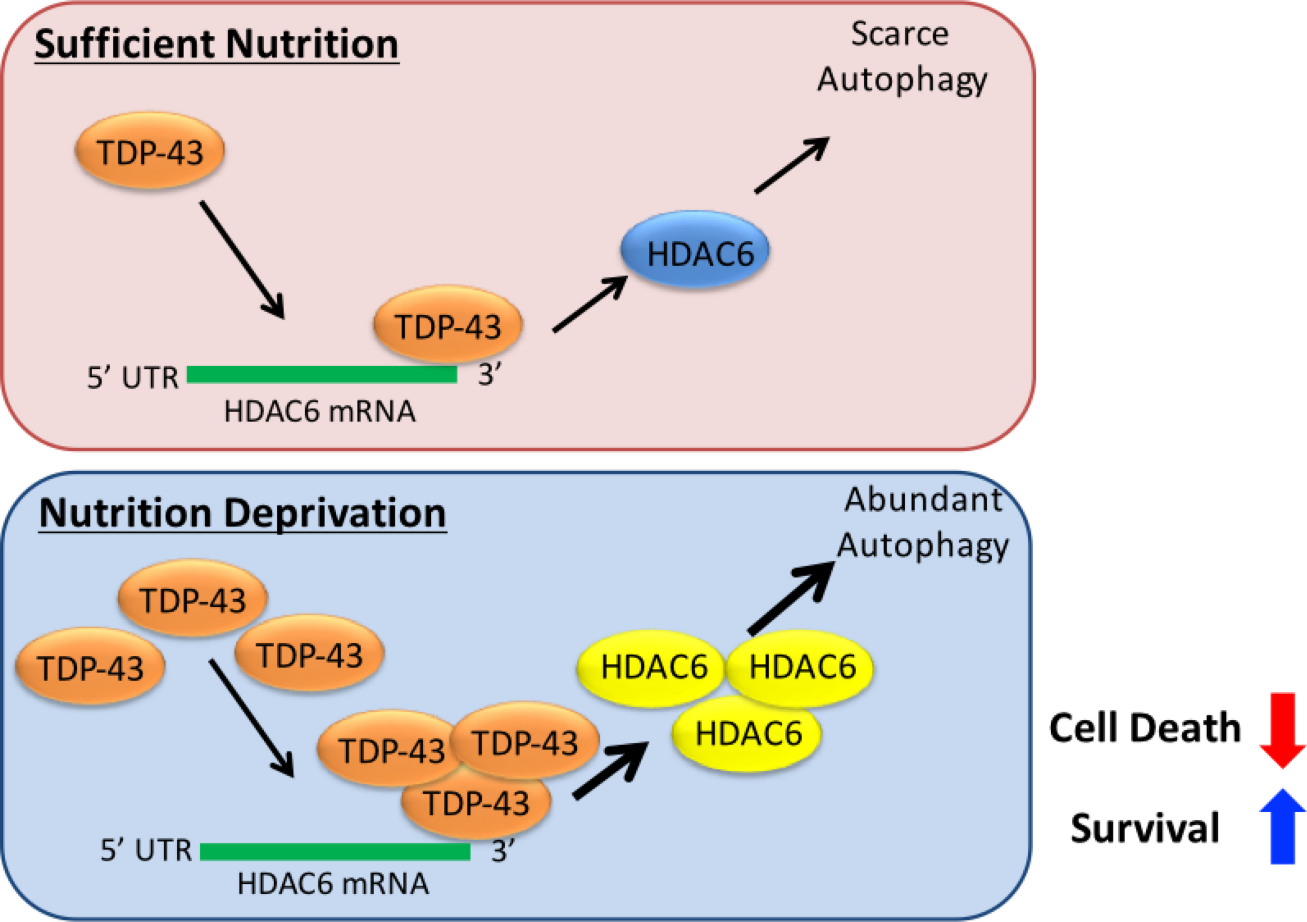
Schematic map of the TDP-43-HDAC6 signal axis in GBM tumorigenesis TDP-43/HDAC6 functions as a stress responsive pathway in GBM tumorigenicity and promotes survival of nutrient-deprived GBM cells via activating autophagy, while inhibition of HDAC6 overpowers the pathway and provides a novel therapeutic strategy against GBM.

To summarize our findings, we demonstrate that: (1) TDP-43 expression is required for tumor progression; (2) TDP-43 modulates autophagy in starved cells, which is in charge of tumor survival under nutrient deprivation; (3) increased stability sustains TDP-43 expression in starved cells; (4) HDAC6 expression mediates anti-apoptotic function of TDP-43. To our knowledge, this is the first demonstration that TDP-43 is involved in tumor progression via autophagy. These findings contribute to our understanding of the potential role of TPD-43 in cancer. In summary, TDP-43/HDAC6 functions as a stress responsive pathway in GBM tumorigenicity and promotes survival of nutrient-deprived GBM cells via activating autophagy (Figure 7).

## MATERIALS AND METHODS

### Cell culture

The human GBM cell line, U87MG, and its derived TDP-43-overexpressed stable cell line were cultured in Dulbecco’s Modified Eagle’s Media (DMEM, Life Technologies Inc., Carlsbad, CA, USA) with 10 % fetal bovine serum (HyClone Laboratories Inc., South Logan, UT, USA), 150g/mL G418 (Life Technologies Inc., Carlsbad, CA, USA), 100 units/mL penicillin, and 100 μg/mL streptomycin (Life Technologies Inc., Carlsbad, CA, USA) under standard culture conditions (37°C, 95 % humidified air and 5 % CO2). Subculturing was performed with 0.25% trypsin-EDTA (Sigma-Aldrich Co. LLC., St. Louis, MI, USA). Media were replenished every two days.

### Colony formation assay

U87MG cells were seeded in 6-well plates (5,000 cells per well) and were incubated for 24 hours. U87MG cells starved in nutrient deprivation condition for 6 days. After 6 days, U87MG cells were refreshed with fresh medium and cultured for 14 days. The cells were fixed with 9% formalin, and stained with 4% trypan blue (w/v) for 20 min. The stained cells were washed using PBS and counted.

### Sphere formation assay

The cells were cultured in tumor sphere medium consisting of serum-free DMEM/F-12, N2 supplement, 10 ng/mL human recombinant bFGF, and 10 ng/mL EGF until sphere formation was observed after about 4 weeks of cultivation.

### Soft agar colony assay

Each well (35 mm) of a six-well culture dish was coated with 1.5 ml bottom agar mixture (DMEM, 10% (v/v) FCS, 0.6% (w/v) agar). After the bottom layer solidified, 1.5 ml top agar-medium mixture (DMEM, 10% (v/v) FCS, 0.3% (w/v) agar) containing 1 × 10^5^ cells was added, and the dishes were incubated at incubator for 4 weeks. Plates were stained with 0.005% (w/v) crystal violet for 1 hour and then a dissecting microscope was used to count the number of colonies.

### Western blotting

Protein samples were prepared with RIPA buffer (Thermo Scientific Inc., Waltham, MA, USA) containing 1% protease inhibitor. Equal weights of total protein were separated by SDS/PAGE. After the proteins had been transferred onto a polyvinylidene difluoride membrane (Millipore, Bedford, MA, USA), the blots were incubated with blocking buffer (1 X PBS and 5% skim milk) for 1 hour at room temperature and then hybridized with primary antibodies overnight at 4°C, followed by incubation with horseradish peroxidase-conjugated secondary antibody for 1 hour at room temperature. The blots were obtained by X-ray film exposure, and the intensities were quantified by densitometric analysis (Digital Protein DNA Imagineware, Huntington Station, NY). The primary antibodies used in this study were anti-Flag (Sigma-Aldrich Co. LLC., St. Louis, MI, USA, F1804), TDP-43 (cell signaling, #3448), cleaved-caspase-3 (cell signaling, #9661), HDAC6 (cell signaling #7558) and beta-actin (sigma, A5316). LC3 (Novus NB100 2220).

### Quantitative real-time PCR (qRT-PCR)

Total RNA was isolated from GBM cells using TRlzol (Life Technologies Inc., Carlsbad, CA, USA) followed by phenol:chloroform purification and ethanol precipitation. Reverse transcription reactions were carried out using SuperScript III reverse transcriptase (Life Technologies Inc., Carlsbad, CA, USA). Oligonucleotides were designed using Primer Express 2.0 (Applied Biosystems, Foster City, CA, USA). Oligonucleotide specificity was computer tested (BLAST, National Center for Biotechnology Information, Bethesda, MD, USA) by homology search with the human genome and later confirmed by dissociation curve analysis. The qRT-PCR was performed with power SYBR Green PCR Master Mix (Applied Biosystems, Foster City, CA, USA) according to manufacturer’s instruction. Signals were detected with 7900HT Fast Real-time PCR system (Applied Biosystems, Foster City, CA, USA). The expression level of each gene was normalized to endogenous beta-actin and experimental control through ΔΔCt method.

### siRNA transfection

The RNAi oligonucleotides targeting TDP-43 (siTDP-43) and the scramble sequence (scRNA) oligonucleotides were all purchased from Ambion. Transient transfection was carried out using INTERFERin siRNA transfection reagent (Polyplus Transfection, Huntingdon, UK) according to manufacturer’s instruction. Cell-based experiments were performed after 2-day incubation.

### Plasmid DNA transfection

Plasmid transfection was carried out using jetPEI DNA transfection reagent (Polyplus Transfection, Huntingdon, UK) according to manufacturer’s instructions.

### Animals and tumor cell transplantation

All procedures involving animals were performed in accordance with the institutional animal welfare guidelines of Taipei Veterans General Hospital. The GBM cell line U87-Flag and U87-TDP-43 were harvested, washed, resuspended in PBS and subcutaneously implanted into the dorsolateral side of the flank region of 8-week-old male BALB/c nude mice (National laboratory animal center, Taipei Taiwan). After 14 days of subcutaneous inoculation tumor volume was measured by calipers.

### Immunohistochemistry staining and immunoblotting (IHC)

Tumor specimens from mice were fixed with 4% paraformaldehyde (Sigma-Aldrich Co., St. Louis, MI, USA). Sections were deparaffinized and rehydrated, and subjected to antigen retrieval by boiling in 10 mmol/L (pH 6) citrate buffer (Sigma-Aldrich Co., St. Louis, MI, USA) for 10 mins. Sections were cooled in PBS for 10 mins before treating with 3% H_2_O_2_. Sample were blocked in 5mg/ml BSA (Sigma-Aldrich Co., St. Louis, MI, USA) for 30 mins before hybridizing with 1/100 diluted primary antibodies TDP-43 (cell signaling, #3448), cleaved-caspase-3 (cell signaling, #9661), HDAC6 (cell signaling #7558) overnight at 4°C. Signals were amplified by the TSA Biotin System (PerkinElmer, Waltham, MA) following the manufacturer’s instruction and the samples were counterstained with hematoxylin. The sections were examined under Olympus BX61 microscope (Olympus Corp., Tokyo, Japan), and three fields of view were randomly selected and photographed for evaluation. The relative staining index (rSI) represents the percentage of positive-expressed cells in the counting region.

### Statistical analysis

The Statistical Package of Social Sciences software (SPSS, Inc., Chicago, IL) was used for statistical analysis. An independent Student’s *t*-test was used to compare the continuous variables between groups. The Kaplan-Meier procedure was used to calculate survival probability estimates. A log-rank test was used to compare the cumulative survival durations in different groups. The statistical significance level was set at 0.05 for all tests.
